# Affecting HEK293 Cell Growth and Production Performance by Modifying the Expression of Specific Genes

**DOI:** 10.3390/cells10071667

**Published:** 2021-07-02

**Authors:** Laura Abaandou, David Quan, Joseph Shiloach

**Affiliations:** 1 Biotechnology Core Laboratory National Institutes of Diabetes, Digestive and Kidney Diseases, NIH, Bethesda, MD 20892, USA; laura.abaandou@nih.gov (L.A.); david.quan@nih.gov (D.Q.); 2 Department of Chemistry and Biochemistry, College of Science, George Mason University, Fairfax, VA 22030, USA

**Keywords:** HEK293, recombinant protein production, cell line engineering

## Abstract

The HEK293 cell line has earned its place as a producer of biotherapeutics. In addition to its ease of growth in serum-free suspension culture and its amenability to transfection, this cell line’s most important attribute is its human origin, which makes it suitable to produce biologics intended for human use. At the present time, the growth and production properties of the HEK293 cell line are inferior to those of non-human cell lines, such as the Chinese hamster ovary (CHO) and the murine myeloma NSO cell lines. However, the modification of genes involved in cellular processes, such as cell proliferation, apoptosis, metabolism, glycosylation, secretion, and protein folding, in addition to bioprocess, media, and vector optimization, have greatly improved the performance of this cell line. This review provides a comprehensive summary of important achievements in HEK293 cell line engineering and on the global engineering approaches and functional genomic tools that have been employed to identify relevant genes for targeted engineering.

## 1. Introduction

The human embryonic kidney cell line (HEK293) was created by transforming human embryonic kidney (HEK) cells with sheared fragments of adenovirus type 5 (Ad5) DNA, immortalizing it. Seventeen percent of the extreme left hand of the Ad5 genome is believed to have been integrated into chromosome 19 of the transformed HEK cells [1]. Since its creation, this cell line has been widely characterized and numerous derivative cell lines possessing unique characteristics have been developed. These cell lines include HEK293T [2], HEK293E [3], HEK293-6E [4], HEK293F and its derivatives HEK293FT and HEK293FTM [2,5,6,7], HEK293S [2], and HEK293H [8]. These derivatives and their properties are summarized in Table 1.

Until two decades ago, the HEK293 cell line was mainly used for transient, small to medium-scale production of research-grade proteins for scientific and pre-clinical studies. Its desirable characteristics have propelled this cell line into a potential producer of biopharmaceuticals [11,12]. These characteristics include suspension growth in serum-free media that enables large-scale production, reproducibility across different batches, rapid reproduction, amenability to different transfection methods, high efficiency of protein production, and—most importantly—its human origin. Five therapeutic proteins produced in HEK293 cells had been approved by the European Medicines Agency or the U.S. Food and Drug Administration for use in humans as of 2015 [13]. This cell line has also been useful in viral vector production as a result of adenoviral DNA in its genome. It has been employed in the production of the influenza virus [14,15], recombinant adenovirus [16], and lentiviruses [17,18]. This cell line is also the preferred host for membrane protein production, where it is most useful as a transient transfection tool for the evaluation of pharmacological properties of multiple receptor subtypes [12,19,20]. 

The HEK293 cell line is of epithelial origin and adherent in nature. It has been adapted to grow in suspension culture in serum-free or chemically defined media and is currently being utilized for recombinant proteins expression in both adherent and suspension platforms. Adherent platforms include roller bottles [21,22,23], multilayered culture systems (e.g., Nunc Cell Factories, Corning CellStacks and Greiner BIO-ONE CELLdisc™) [24,25,26,27,28], and fixed-bed bioreactors [27,29]. Suspension platforms include batch, fed batch and continuous processes in bioreactors. 

Due to its human origin, HEK293 is better suited for production of biotherapeutics for human use since it produces protein with native post-translational modifications (PTMs). Non-human cell lines, such as the Chinese Hamster ovary cells (CHO), have been shown to implement incomplete humanized PTMs and can introduce potentially immunogenic non-human glycosylation patterns, most notably α-gal and NGNA [30,31]. PTMs, especially glycosylation patterns, affect the pharmacokinetics and pharmacodynamics of recombinant biotherapeutics. Although extensive comparative analyses of the PTMs on recombinant proteins produced in numerous cell lines have concluded that the suitability of a cell line for producing human therapeutic proteins should be determined on a case-by case basis [32,33], the HEK293 cell line has been shown to be especially efficient in tyrosine sulfation and glutamic acid γ-carboxylation when compared to other cell lines [34,35,36,37,38]. The impact of the host cell line on glycan profile has been comprehensively reviewed by Goh and Ng [39]. However, clinical experience with the HEK 293 cell line is not as extensive as for non-human other cell lines, although experience is growing, and there is potential susceptibility to human viral contamination due to the absence of species barrier. 

Other human cell lines such as the HT-1080, produced from a fibrosarcoma with an epithelial-like phenotype [11] and the PER.C6 cell line created from human embryonic retinal cells, immortalized via transfection with the adenovirus E1 gene [40], have comparable growth, transfectability, production and safety profiles to the HEK293 cell line and produce complex human proteins with humanized PTMS [13]. However, the HEK293 cell line was used more extensively in different research topics and has numerous derivative cell lines which greatly expands the repertoire of recombinant proteins that it can efficiently produce.

Process and media parameters, such as expression vector, growth media, transfection agent and culture conditions, have been optimized to improved recombinant protein yields from HEK293. Notable innovative approaches include 3D collagen microsphere culture system [41]; the establishment of a glutamine-ammonia ligase (GLUL) mediated gene selection system [42]; glutamine synthase (GS) mediated selection/amplification system, and a dihydrofolate reductase (DHFR) amplification system [43,44], synonymous with the CHO system [45]. Growth and media optimization have led to the development of several formulations of chemically defined growth media and animal-derived component free media additives tailored to HEK293 cell growth and protein expression [46]. Some of the most widely utilized serum-free chemically defined media include ThermoFisher’s FreeStyle™ 293, CD 293, 293 SFM II and Expi293™ Expression Medium, Millipore Sigma’s EX-CELL^®^ 293 Serum-Free Medium for HEK 293 Cells, and Cytiva’s HyClone SFM4Transfx-293 media.

Several transfection reagents have been optimized for use in transfection of HEK293 with plasmid DNA. For example, the Lipofectamine 3000 reagent by Thermo Fisher Scientific is the superior—but cost-prohibitive—option [47], while polyethylenimines (PEI) is the more cost-effective option [48]. Expression vector optimization identified the human cytomegalovirus promoter (CMV) as the most effective promoter for the expression of heterologous proteins in this cell line [49]. Culture parameters, including temperature and carbon dioxide concentration, [50] have also been optimized. Cytostatic agents such as sodium butyrate, trichostatin A, valproic acid, and dimethyl sulfoxide [51], have been used to enhance production. The implementation of these strategies to boost protein yields has resulted in product yields of up to 100–200mg/L in adherent systems, while suspension systems have yielded up to 140–600mg/L of recombinant protein [52,53]. 

Yet, despite these significant improvements in recombinant protein and virus production, this cell line continues to lag behind the CHO cell line in growth capacity (Maximal cell density of 3-5 × 10^6^/mL for HEK293 vs. 1-2 × 10^7^ for CHO) [42], cultivation time (HEK293 doubling time 33 h vs. 14-17 h for CHO), and product yield in stable production systems- up to 4g/L for CHO cells vs. 600mg/L for HEK 293 cells [54]. Consequently, some innate characteristics of HEK293 cells have been modified, resulting in higher recombinant protein yields, efficient nutrient utilization, and more homogenous glycosylation. This review will discuss the genetic engineering strategies, functional genomics and bioinformatic approaches that have been employed to improve the quality and quantity of recombinant protein production in the HEK293 and its derivative cell lines. 

## 2. Strain/Genetic Engineering for Enhanced Recombinant Protein Expression in the HEK293 Cell Line

**Figure 1:**
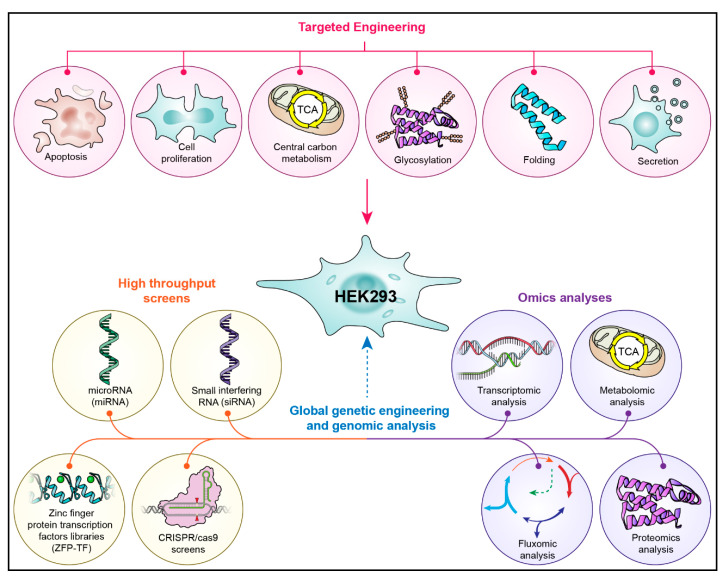
Engineering the HEK293 cell line for improved culture performance. In targeted engineering, genes involved in processes directly affecting protein expression such as apoptosis, cell proliferation, central carbon metabolism, glycosylation, protein folding, and secretion are modified. Global engineering encompasses high throughput screens using RNA interference (miRNA and siRNA), Zinc Finger protein transcription factor libraries, and CRISPR/Cas 9 libraries, and omics analysis such as transcriptomic, proteomic, metabolomic, and fluxomic analyses that are utilized to discover novel engineering targets.

Although media and process optimization efforts have resulted in significant improvements in recombinant protein titers, the cell’s inherent biology is the ultimate limiting factor of the amount of recombinant protein that it can produce. The introduction of mutations in chromosomes, such as nucleotide substitutions, gene deletions or insertions, and the overexpression of homologous or heterologous genes can affect the expression of target proteins. Specific genes in cellular pathways involved in protein biogenesis can be temporarily or permanently modified in a targeted manner by using RNA interference, Zinc Finger Nucleases (ZFNs), transcription activator-like effector nucleases (TALENs) and the clustered regularly interspaced short palindromic repeats, CRISPR-associated protein 9 (CRISPR-Cas9) gene editing systems. Global genetic or strain engineering and whole genome analysis tools, such as random mutagenesis, RNA interference screens, whole genome fluxomic, metabolomic, proteomic, and transcriptomic analyses, have also been employed to identify novel target molecules involved in protein biogenesis. These engineering and analysis strategies are summarized in Figure 1.

### 2.1. Targeted Genetic Engineering Approaches

An efficient approach for the modification of cell lines is targeted engineering of biosynthetic bottlenecks. This approach involves the modification of genes with known effects on protein synthesis, protein folding (including the implementation of PTMs), secretion, and degradation. Typically, inactivating mutations are introduced into genes that negatively impact recombinant protein expression, while genes with a positive effect on recombinant protein expression are overexpressed. Engineering gene targets will be reviewed under the following categories: cell proliferation and apoptosis; central carbon metabolism, glycosylation, protein folding, and secretory pathways. 

#### 2.1.1. Genes Involved in Cell Proliferation and Apoptosis

The comparatively long doubling time (~33 h) of HEK293 cells in suspension culture (CHO cell doubling time is 14–17 h) unfavorably affects the rate of recombinant protein production. Cells entering late log and early stationary phases are experiencing decreasing growth rates and ultimately cell death by apoptosis and necrosis [55]. The goal of engineering cell proliferation and apoptosis is to increase culture longevity and maximal cell density, while maintaining per cell production, thereby enhancing recombinant protein and viral titers. This goal has been primarily achieved by altering the cell growth rate and/or the cell death rate. 

Proliferation: Growth rate has been modified by either slowing or accelerating the transitions between different cell cycle phases. Increasing the growth rate by overexpressing genes that promote cell cycle transitions, such as the G1/S promoting cyclin dependent kinase like 3 protein (*CDKL3*), cytochrome c oxidase subunit, Cox15 (*COX15*) [56], components of the eukaryotic initiation factor 3 complex, *EIF3I* and *EIF3C* [57], and the acidic fibroblast growth factor (*FGF1*) [58], has successfully improved total recombinant protein expression. Slowing growth rate at the G0/G1 or the G2/M phases, where cells are generally larger and more metabolically active, by overexpressing genes that inhibit the transitions between the phases such as the cyclin-dependent kinase inhibitors (*CDKN*) *1B* [59], *2C* and *1A* [58] has proven to be effective for improving specific production in HEK293 cells. Significant improvements in recombinant protein expression (up to 5.9-fold) have been achieved using these strategies. The overexpression of *CDKN1A, CDKN2C* and *FGF1*, combined with rational vector design and valproic acid treatment resulted in up to 27-fold improvement (from 40 mg/L to 1.1 g/L), in recombinant immunoglobulin G (IgG) expression in a transient system [58]. It is noteworthy to emphasize that cell proliferation engineering is a balancing act; extremely high proliferation rates can lead to reduced recombinant protein expression due to cellular resources being disproportionately used for proliferation, while the increased specific productivity achieved by slowing down growth rate might not be enough to compensate for the loss in cell density. Cell proliferation genes that have been engineered in the HEK293 cell line are listed in Table 2. 

Apoptosis: Creating cells resistant to apoptosis can potentially increase culture longevity, viable cell numbers, and recombinant protein expression. Apoptosis is under the control of pro- and anti-apoptotic factors whose expression is triggered by nutrient deprivation, low oxygen levels, high osmolarity, or DNA damage. The onset of apoptotic cell death has been effectively delayed in HEK293 cells by down-regulating pro-apoptotic genes, as in the double knock out of effector caspase activators, *BAX* and *BAK* [60], and the quadruple knock out of the executioner caspases Caspase3, Caspase6, Caspase7 and the Allograft inflammatory factor 1 (*AIF1*) genes [61]. Another approach is to upregulate anti-apoptotic genes, such as the overexpression of the X-linked inhibitor of apoptosis (*XIAP*) [62,63,64], the Nuclear factor erythroid 2-related factor 2 (*NRF2*) [65], and the anti-apoptotic B-cell lymphoma 2 (*BCL2*) genes [63,66]. These modifications have resulted in up to 8-fold higher numbers of viable cells and up to a 40% and 53% increase in recombinant protein levels viral vector titers, respectively. Similarly, the expression of the heterologous viral apoptosis inhibitor, *CrmA* in HEK293 cells, conferred protection against apoptosis insults, such as spent medium and Sindbis virus infection, and by the chemotherapeutic reagent etoposide [64]. Apoptotic genes that have been modified in the HEK293 cell line are summarized Table 3. 

#### 2.1.2. Genes Associated with Central Carbon Metabolism 

HEK293 is inefficient at metabolizing glucose [67,68,69,70], this characteristic forces the cell to utilize large amounts of glutamine for energy generation. The by-products of the inefficient glucose metabolism and glutamine utilization are lactate and ammonia, which cause premature cell death and low recombinant protein titers [71,72]. High ammonia concentrations also negatively affect protein glycosylation [73,74,75,76]. As a result, metabolic engineering of HEK293 cells has been concentrated on improving the central carbon metabolism. This has been achieved by restoring the link between glycolysis and the tricarboxylic acid cycle through overexpression of the pyruvate carboxylase (*PC*) gene [77,78,79,80], and by the knockdown of the pyruvate dehydrogenase kinase gene (*PDK*), alongside its activator, the hypoxia inducible factor 1 (*HIF1*) gene [81]. These modifications resulted in increased cell density, decreased lactate and ammonia production, altered amino acid utilization, enhanced recombinant viral titers of up to 30-fold, enhanced recombinant protein expression, and improved glycosylation. Process optimization strategies, such as maintaining constant glucose concentration throughout the culture [79] or substituting glutamine with alternative substrates, such as pyruvate, α-ketoglutarate or glutamate, or eliminating glutamine addition [78], have further enhanced the effects of PC overexpression. The different approaches and outcomes of these modifications are shown in Table 4. 

#### 2.1.3. Genes Involved in Protein Maturation and Processing 

In addition to the production of proteins for therapeutic use, the HEK293 cell line is used for the expression of proteins with proper post-translational modifications (PTMs), such as glycosylation, for structural biology studies [82]. Efficient producing cell lines that perform proper PTMs are especially important for recombinant proteins since these bioproduction cell lines are generally not the natural in vivo producers of these proteins, such as IgG production in HEK293 cells, which are of nephritic origin. Furthermore, the expression of these proteins is driven by strong promoters, that can overwhelm the cell’s folding and secretory machinery, resulting in a secretory backlog or Endoplasmic Reticulum (ER) stress. Quality control mechanisms such as ER-associated degradation (ERAD) which targets misfolded proteins of the ER for ubiquitination and subsequent degradation by the proteasome, could also be overwhelmed. The stresses to this machinery could cause the accumulation of improperly folded proteins in the cell, or a secretory backlog which could result in a general attenuation of translation and cell death by apoptosis. 

Glycosylation: Despite its human origin, challenges exist when expressing glycosylated recombinant proteins in HEK293 for biotherapeutic purposes or structural biology studies, notably the introduction of heterogenous N-glycan glycosylation patterns. Homogenous glycosylation patterns on biotherapeutics are important beyond maintaining protein quality and reproducibility; they also affect folding, intracellular transport, stability, interactions, and clearance [83,84]. Homogeneous glycoproteins are also important for structural studies, in particular for the improvement of the X-ray diffraction properties of *N*-glycosylated proteins [85]. Consequently, most glycosylation engineering in HEK 293 has focused on improving glycan pattern homogeneity by targeting enzymes involved in glycan processing-glycosyltransferases and glycosidases. The three major types of N-glycans in order of complexity are the high mannose type, the hybrid type, and the complex type. Genes encoding enzymes catalyzing reactions that increase glycan heterogeneity, such as the Alpha-1,3-Mannosyl-Glycoprotein 2-Beta-N-Acetylglucosaminyltransferase-GnTI (*MGAT1*) [86,87], the Golgi mannosidases alpha-1,2-mannosidase IA and alpha-1,2-mannosidase IB (*MAN1A1* and *MAN1A2*) [78], alpha-mannosidase 2 and alpha-mannosidase 2x (*MAN2A1* and *MAN2A2*) and the Alpha-(1,6)-fucosyltransferase (*FUT8*) [88], the endoplasmic reticulum mannosidase I (*MAN1B1*) [89], and the Golgi α1,2-mannosidase-I (*MAN1C1*), have been knocked out, while those that hydrolyze complex glycans, such as the endo-β-N-acetylglucosaminidase, *endoT^8^* from the fungus *Hypocrea jecorina* (fused to the Golgi targeting domain of the human β-galactoside-α-2,6-sialyltransferase 1), have been overexpressed [90]. Research groups have targeted different combinations of these enzymes resulting in improved homogeneity of glycosylation patterns on expressed recombinant glycoproteins in HEK293. For example: the 293SGlycoDelete cell line, deficient in the GnTI enzyme and overexpressing the *endoT^8^* gene, engineered by Meuris and colleagues [90] has been utilized to produce proteins with homogenous mature-like trisaccharide glycan stubs for crystallogenesis [85]. Quadruple and quintuple knockout cell lines, deficient in the *MAN1A1, MAN1A2, MAN1B1, MAN1C1* and *MGAT1* genes, produce proteins expressing only high-mannose-type N-glycans [86]. A double *MAN2A1* and *MAN2A2* knockout cell line mainly produces hybrid type N-glycans, while the triple *MAN2A1*, *MAN2A2* and *FUT8* knockout cell line only produced hybrid type glycans without core fucosylation [88] Sialylation of recombinant proteins, which affects protein activity and in vivo circulatory half-life, has been improved by overexpressing genes encoding the sialyl transferases ST3GalII, ST3GalIV and ST6Gal1 [91,92]. These engineering targets are summarized in Table 5. 

Protein folding and other protein modifications: The folding machinery of producing cell lines is challenged by unusually high quantities of recombinant proteins that are oftentimes not native to the cell. The accumulation of misfolded proteins in the ER may induce the unfolded protein response (UPR), which eventually triggers apoptosis if left unresolved [93,94]. The aim of protein folding engineering is to prevent the induction of the UPR, or to quickly resolve it by overexpressing endoplasmic reticulum (ER) resident folding factors, such as chaperones, cochaperones, peptidyl prolyl *cis/trans* isomerases (PPIases), oxidoreductases, protein disulfide isomerases (PDI), and glycan-binding proteins. The X-box binding protein (*XBP1*), a transcription factor which induces genes involve in the UPR, has also been targeted for engineering. The simultaneous overexpression of the PDI, Protein Disulfide Isomerase Family A Member 2 (*PDIA2*), the chaperone heat shock protein family A (Hsp70) member 5 (*HSPA5*), and the *XBP1* resulted in improvements of both volumetric and specific gamma retroviral vector production of up to 97% and 92%, respectively [66]. The production of difficult to express proteins has been enhanced by their co-expression with their molecular chaperones. For example, the difficult-to-express recombinant α-7 nicotinic acetylcholine receptor has been successfully co-expressed with its molecular chaperone, resistance to inhibitors of cholinesterase 3 (*RIC3*) [95].

Gamma-carboxylation (γ-carboxylation) of a cluster of glutamate residues near the amino termini of most clotting factors is required for these proteins to bind Ca++ and function efficiently in blood clotting. The efficiency of this process in HEK293 cells has been increased by overexpressing the rat Vitamin K epOxide Reductase Complex (*VKORC1*) subunit 1 [96] gene and downregulating the inhibitor of γ-carboxylase, calumenin (*CALU*) [97]. These engineering targets are summarized in Table 6. 

Secretory pathways: Secretion can be limiting factor for host cell productivity, especially in high producing cell lines [98,99,100,101,102,103,104,105]. A key rate limiting step of the secretory pathway is the fusion of vesicles with the target membranes, which is catalyzed by SNARE (soluble NSF (N-ethylmaleimide-sensitive factor) receptor) proteins and regulated by Sec1/Munc18 (SM) proteins [106,107]. Overexpression of members of both the SNARE and SM protein families has been employed to increase the secretory capacity of HEK293. Overexpressing genes encoding the synaptosome-associated protein of 23 KDa (SNAP-23) and the vesicle-associated membrane protein 8 (VAMP8) SNARE proteins, together with the SM proteins Syntaxin-binding protein 1,(Munc18b) and yeast suppressor of loss of YPT1 protein 1 (Sly1) [108], significantly improved the secretory capacity of HEK293. The result was up to a 2-fold increase in both secreted recombinant protein and lentiviral titers. Similarly, the production yield and functionality of Exosome-Associated AAV has been enhanced by up to 26% by the overexpression of the tetraspanin *CD9*, which enhances exosome biogenesis, [109]. These engineering targets are summarized in Table 7. 

### 2.2. Global Genetic Engineering and Genomic Analysis Approaches

In addition to the targeting of known genes and pathways, screening and omics analysis approaches have also been utilized in the quest to improve HEK293 culture performance. Such studies employ functional genomic tools to identify novel genes and pathways that limit or enhance the expression of recombinant proteins in HEK293. Identifying genes using this approach obviates the need for *a priori* hypotheses or knowledge regarding potential bottlenecks in recombinant protein expression. Global genetic engineering approaches typically involve screening a cell line expressing an easily quantifiable reporter protein, such as luciferase or green fluorescent protein (GFP), against an array of gene-specific factors. The screening is followed by evaluation of the effect of individual factors on the expression of the reporter protein. These factors may include RNA species, such as siRNA, miRNA, and shRNA, that bind to and silence homologous RNA sequences, and DNA-binding molecules, such as single guide RNAs(sgRNA) of the CRISPR/Cas9 system, and zinc finger proteins, that bind to and modify DNA in a sequence-specific manner. Global engineering approaches used to query bioproduction in HEK293 cells include high throughput RNA interference screen, omics analysis, randomized zinc finger protein transcription factors, and genome wide CRISPR/Cas9 screens. These strategies are summarized in Table 8.

#### 2.2.1. High Throughput RNA Interference (RNAi) Screens

RNA interference is a conserved biological process in eukaryotes that regulates the expression of protein coding genes at the post-transcriptional level. This process, which is mediated by two types of small RNA molecules—microRNA (miRNA) and small interfering RNA (siRNA)—is being exploited to selectively silence specific genes for functional studies. High throughput RNAi, which utilizes this process on a larger scale, has been proven to be a fast and unbiased tool for identifying genes involved in cellular processes such as cancer, cell survival, and recombinant protein expression. In this approach, a specific property of the targeted cells is measured while the cells are exposed to a library of microRNA or siRNA. 

High throughput micro-RNA (miRNA) screens: Micro RNAs are small, non-coding RNA molecules found in most organisms that function via base-pairing with complementary sequences within mRNA molecules, silencing gene expression. In a 2015 study, our group carried out a mimic microRNA (miRNA) screen to identify miRNAs that can potentially improve HEK293 cell line performance, using the neurotensin receptor as the reporter protein. Cells were screened with a library comprised of 875 human miRNA mimics that resulted in the identification and validation of hsa-miR-22-5p, hsa-miR-18a-5p, hsa-miR-22-3p, hsa-miR-429, and hsa-miR-2110 as potential candidates for improved recombinant protein expression. The treatment of cells with each of these miRNAs led to improvements in expression of membrane neurotensin receptor (NTSR), cytoplasmic firefly luciferase (FLuc), and secreted glypican −3 hFc-fusion protein (GPC3), ranging from 1.1- to 3-fold [111]. In follow-up studies, the gene targets of miRNA-22-3b were identified, and the homeodomain-interacting protein kinase 1 (*HIPK1*) gene was determined to be the most significantly involved in improving recombinant protein expression stimulated by this miRNA. The knockdown of the *HIPK1* gene using siRNA resulted in a 3.2-fold increase in FLuc expression and a 2.3-fold increase in GPC3 expression [122]. A stable cell line in which the *HIPK1* gene was knocked out by using CRISPR/Cas9 genome editing had up to a 4.7-fold improvement in FLuc expression, while the stable overexpression of miRNA-22-3b resulted in a 2.4-fold improvement in luciferase activity [113]. 

High throughput small interfering RNA (siRNA) screens: Small interfering RNA (siRNA) is a class of double-stranded non-coding RNA molecule, typically 20–27 base pairs in length, similar to miRNA, and operating within the RNA interference (RNAi) pathway. These molecules interfere with the expression of specific genes through complementary nucleotide sequences by degrading mRNA after transcription, thereby preventing translation. Synthetic siRNA molecules that silence almost every gene in the genome have been developed for the human and other relevant genomes. Lwa and colleagues executed a targeted high throughput siRNA screen using a panel of 153 siRNA pairs that targeted 153 representative genes involved in the unfolded protein response (UPR), transcription, and cell cycle regulation using HEK293 cells transiently expressing the secreted Gaussia luciferase (Gluc), and gene hits confirmed in erythropoietin (EPO) expressing cells. The KCTD2 and CEBPG genes whose knockdown negatively affected the expression of Gluc were identified as potential engineering targets. Their overexpression enhanced transient production of both Gluc and EPO by 2- to 4-fold in HEK293 cells [114]. 

On a much larger scale, our group performed a high throughput genome-wide siRNA screen where 21,585 genes were individually silenced with six different siRNAs each in HEK293 cells constitutively expressing the Fluc reporter gene and confirmed in cells expressing secreted GPC3, neurotensin receptor type I and the serotonin transporter. The knockdown of *INTS1, HNRHPC, OAZ, CASP8AP2* and *PPP2R1A* consistently improved the expression of at least two reporter proteins tested by up to 72% [115]. In follow-up studies, the CRISPR/Cas9 knockout of the ornithine decarboxylase antizyme1 (*OAZ1*) resulted in a 5-fold improvement in luciferase production and a 2.5-fold improvement in transient specific secreted alkaline phosphatase (SEAP) production [116], and knockout of the caspase 8 associated protein 2 (*CASP8AP2*) gene resulted in a 7-fold increase in specific luciferase production and a 2.4-fold increase in transient specific SEAP production [117]. 

#### 2.2.2. Genome-Wide CRISPR/Cas9 Screens

Genome-wide CRISPR/Cas9 screens have emerged as a powerful tool for performing large-scale loss-of-function screens, with low noise, high knockout efficiency, and minimal off-target effects. This approach utilizes the CRISPR-Cas9 gene editing system, coupled with libraries of single guide RNAs (sgRNAs), which are designed to target every gene in the genome. The main advantages of genome-wide CRIPSR screens over RNA interference screens is the ability to achieve highly efficient and complete protein depletion [123] without the off-target issues seen with RNAi screens. RNAi is also limited to transcripts, whereas Cas9: sgRNAs can target elements across the entire genome, including promoters, enhancers, introns, and intergenic regions. Although this highly effective tool has not yet been used in the discovery of engineering targets in the HEK293 cell line, it has been used to identify genes essential for recombinant protein expression in other cell lines. For example, novel genes involved in protein secretion in HeLa cells [124] and novel genes involved in glycosylation in a human bladder cancer cell line [125] have been identified using this tool.

#### 2.2.3. Randomized Zinc Finger Protein Transcription factors (ZFP-TF) Libraries 

ZFP-TF libraries are libraries of zinc finger proteins (ZFPs) with distinct DNA-binding specificities fused to either a transcriptional activation or repression domain that activate or repress specific genes. ZFP-TFs have been screened for various phenotypic changes, such as drug resistance, thermo-tolerance or osmo-tolerance in yeast, and differentiation in mammalian cells [126]. Park and colleagues exploited the capabilities of ZFP-TF libraries for random activation or repression of endogenous genes to improve the yields of industrially relevant recombinant proteins, human growth hormone (hGH) from *S. cerevisiae* and secretory alkaline phosphatase (SEAP) from HEK293 cells. By co-transfecting a plasmid-encoding SEAP with randomly chosen plasmids from a ZFP-TF library, and screening individual clones for SEAP production, they identified a single ZFP-TF fused to an activator which improved SEAP production 5-fold, and two ZFP-TF fused to repressors which improved SEAP expression 2- to 3.5-fold. The activator-fused ZFP-TF acted synergistically with either repressor-fused ZFP-TF. These results were reproducible in other human cell lines [127].

#### 2.2.4. Omics Analysis

Another strategy to identify engineering targets for improved expression is omics analysis, which includes genomic, transcriptomic, metabolomic, proteomic and fluxomic. Identification of relevant genes and pathways is done by comparing the omics of homologous (or sometimes heterologous) cell lines exhibiting disparate production characteristics to uncover differentially expressed genes, abundant proteins and consumed or secreted metabolites (both in quantity and rate of consumption/secretion), that could provide insight into potential production bottlenecks. In 2012, Dietmair and colleagues performed a multi-omics study in which they compared the transcriptome, metabolome and fluxome of a stable HEK293 cell line producing a heavy chain variable region fused to the Fc region of human IgG (dAb-Fc), with the non-producing parental cell line [118]. Fluxomic analysis, which quantifies the rates of metabolic reactions within the central carbon metabolism, revealed lower glycolytic flux in the producer cultures, that was associated with the reduced glucose uptake. The authors speculated that the highly downregulated (6.3-fold) fatty acid binding protein 5 (*FABP5*) gene and the highly upregulated (66-fold) resistin-like β (*RETNLB*) gene are associated with the reduced glucose uptake of the producer cell line. Additionally, a pyruvate carboxylase (PC) flux was present in the producer, but not in non-producer cultures. *PC* overexpression has been previously employed to improve the metabolic efficiency of HEK293 and CHO producer cell lines [80,128]. Transcriptomic analysis revealed downregulation of genes involved in transcription and translation in the producer cell line; at the same time, the producer culture showed upregulation of several genes related to ER stress in including X-box binding protein (*XBP1*), *HSPA5* and *HERPUD1*, suggesting that the UPR (unfolded protein response) was activated and protein folding capacity may limit recombinant protein production in this cell line. Heterologous expression of *XBP1* has previously been shown to increase the specific therapeutic antibody productivity of CH-DG44 cells in inoculum suspension cultures [99], and was recently shown to improve gamma retroviral vector production in HEK293 cells [66]. Although no follow-up studies have been carried out based on the reported results, the observation that some of the genes identified were previously validated as engineering targets emphasizes the relevance of such studies. It would be useful to validate novel gene hits identified in this study as potential engineering targets for improved recombinant protein production in HEK293 cells. 

In another transcriptomic analysis study, Rodrigues and colleagues compared two human cell lines of different genetic backgrounds, producing a murine leukemia virus-based vector- a high producer Te Fly Ga 18 with a low producer 293 FLEX (derived from the Te 671 and HEK 293 cell lines, respectively). Analysis of metabolic pathways showed upregulations in branched chain amino acid catabolism, de novo nucleotide synthesis, polyamine biosynthesis, glutathione mediated detoxification, and folate transformations, in the high producer cell line. By modifying the identified pathways using media manipulation (supplementation with nucleosides, amino acids, antioxidants, polyamines and reduced glutathione), it was possible to increase the specific productivity of the virus-based vector by up to 600% in the low producing 293 FLEX cell line [119].

In a 2020 proteomic study, Lavado-Garcia and colleagues compared the proteome of HEK293 when growing without transfection, when transfected with an empty plasmid, and when transfected with Gag coding gene producing Gag VLPs, to identify bottlenecks in growth, transfection and VLP production. Identified bottlenecks in transfection and growth included pathways downregulated in transfected vs. untransfected cells, such as lipid biosynthesis, intracellular protein transport, glycosphingolipids metabolism, calcium regulation, oxidant detoxification, xenobiotic metabolism, peptidase activity and DNA detoxification pathways. Endocytosis, late endosomal-related pathways and members of the endosomal sorting complex required for transport (ESCRT) (ESCRT-1 complex, neural precursor cell expressed developmentally downregulated 8 (NEDD8), and neural precursor cell expressed developmentally downregulated 4-like (NEDD4L) proteins), which were downregulated in VLP producing vs. empty plasmid- transfected cells, were identified as bottlenecks in VLP production [120]. 

In a follow-up study carried out by the same group, overexpression of members of the endosomal sorting complex, (*NEDD8*, *NEDD4L* and *CIT*), and the glycosphingolipid precursor enzyme UDP-Glucose Ceramide Glucosyltransferase (*UGCG*) in HEK293 cells resulted in respective 1.5-, 3.3-, 2.9- and 2.4-fold improvements in VLP production, accompanied by a 51-61% increase in budding efficiency, and a 17% increase in transfection efficacy. In addition, shRNA Knockdown of the Gag-binding protein 2’,3’-Cyclic Nucleotide 3’ Phosphodiesterase gene, *CNP*, which inhibits viral replication, resulted in a 2.7-fold increase in VLP production and a 36% increase in budding efficiency [121].

## 3. Conclusions

Although the HEK293 cell line trails non-human cell lines such as CHO and NSO, in recombinant protein production and culture performance [13], it is our opinion that this cell line has a place in the production of specific bioproducts. These cells, in addition to their ability to create native PTMs, efficiently execute tyrosine sulfation and gamma carboxylation which makes them the preferred host for recombinant clotting factors and natural anticoagulants production [13]. The cells also gained regulatory approval and have a place as producer of difficult-to-express antibody fragments or artificial scaffolds, which are forecasted to be the future of antibody therapy [129]. In addition, their use in production of gene therapy vectors will only increase as shown by the number of studies and resources directed towards understanding and improving gene therapy vector production in recent years [130]. The author’s opinion is that rational engineering strategies targeting bottlenecks in proliferation, carbon metabolism, protein processing and maturation with discovery of engineering targets using functional genomics tools have and will continue to enhance the production efficiency of the HEK293 cell line. 

Among the targeted engineering strategies discussed in this review, glycosylation is the most critical. Obtaining homogenous glycosylation patterns is practically impossible without genetic manipulation or chemical treatment of the cell line [84]. Two HEK293 cell lines with improved glycosylation profiles are currently in use; the commercially available HEK293SG (HEK293SGnTI^-^ (ATCC^®^ CRL-3022^™^), in which the N-acetyl-glucosaminyltransferase I (GnTI) gene has been knocked out and lacks complex N-glycans, and its derivative cell line HEK293SGGD (293SGlycoDelete (RRID:CVCL_6E38), which expresses a heterologous Golgi endoglycosidase for secretion of de-glycosylated glycoproteins. In addition, treatment of cells with small molecule inhibitors such as kifunensine, that targets α-mannosidase I and swainsonine, that blocks α-mannosidase II, results in relatively simple and chemically uniform glycans [82]. 

The majority of the currently approved therapeutics are being produced in derivative cell lines, underscoring the relevance of their unique properties; the recombinant FVIIIFc and recombinant FIXFc (ALPROLIX^®^, 2014; ELOCTATE^®^, respectively) in HEK293-H, the Human-cl rhFVIII (NUWIQ^®^) produced in HEK293F, the Glucagon-1-like peptide (GLP-1) Fc fusion protein (dulaglutide, TRULICITY^®^) produced in HEK293 EBNA, while the currently withdrawn drotrecogin alfa (XIGRIS^®^) was produced in the parental HEK293 cell line [13].

On the VLP and viral vector production front, parental HEK293 and derivatives such as HEK 293T and HEK293FT are the preferred hosts, the latter two due to the presence of the SV40 large T-antigen in their genomes that increases their viral production efficiency. Virus-like particle (VLP for vaccine generation) produced in HEK293 cell line include Rabies virus G protein VLP, Influenza virus A/Puerto Rico/8/34 (H1N1) VLP, Hepatitis B virus small surface antigen (HBaAgS) VLP, Human scavenger receptor class B, member 2 (SCARB2) VLP; Lentiviral vectors for gene therapy: Kymriah™ (Lymphoblastic leukemia) and ZyntegloT™ (Beta-thalassemia); AAV gene therapy vectors: Glybera™ (Lipoprotein lipase deficiency), Luxturna™ (Leber congenital amaurosis), and Zolgensma™ (Spinal muscular atrophy type 1); retroviral gene therapy vectors: Rexin-G™ (Pancreatic cancer), Strimvelis™ (Adenosine deaminase—immunodeficiency), Zalmoxis™ (Leukemia) [131].The choice of HEK cell line for producing viral vectors is usually more straightforward compared to the production of recombinant proteins. For most recombinant proteins produced in the HEK293 cell line and its derivatives, the choice of cell line is assessed on a case-by-case basis, and it is not uncommon for researchers to test different cell lines for productivity before deciding. Notwithstanding, the HEK293H and HEK293F cell lines with their superior transfectivity, higher productivity, and faster growth to high densities in chemically defined media are currently the choice for large scale recombinant protein production. 

The HEK 293 cell line, along with a few other human cell lines, currently serves as a valuable niche for the production of biotherapeutics that require human PTMs. It is also the cell line of choice for the production of recombinant gene therapy vectors. Whether or not this cell line will become as widely used as the CHO cell line in biotherapeutic production will depend on the continued technological achievements and research investments in further optimizing this cell line. 

## Figures and Tables

**Table 1:** HEK293 cell line derivatives.

HEK 293 Variant	Derivation	Commercially Available?	Desired Characteristic(s)	Year of Derivation
HEK293	Transformation of Human embryonic kidney cells with sheared fragments of adenovirus type 5 (Ad5) DNA, selected for immortalization.	Yes	Parental HEK293 cell line	1973
HEK293S	Adapted for suspension growth	Yes	Grows in suspension in modified minimal Eagle’s medium	1984
HEK293T	Stable transfection of the HEK 293 cell line with a plasmid encoding a temperature-sensitive mutant of the SV40 large T antigen	Yes	Amplification of vectors containing the SV40 ori, considerably increasing the protein expression levels during transient transfection.	Before 1985
HEK293FT	Fast-growing variant of HEK293T. Expresses the SV40 large T antigen from the pCMVSPORT6TAg.neo plasmid.		Designed for lentiviral production.	
HEK293F	Cloned from the HEK293 cell line and adapted to commercial medium	Yes	Fast growth and high transfectivity. Growth in chemically defined medium	2014
HEK293H	Cloned from HEK293 to select a clone with good adherence during plaque assays. Later adapted to growth in serum-free medium (SFM) (GIBCO 293H)	Yes	Fast growth in SFM, good adherence during plaque assays, superior transfection efficiencies and a high level of protein expression	1993
HEK293E(EBNA)	Expresses the EBNA-1 protein for episomal replication of oriP harboring plasmids	Yes	Amplification of vectors containing oriP, considerably increasing the protein expression levels during transient transfection.	
HEK2936E	This cell line is transfected with EBNA1t, a truncated version of EBV EBNA1 lacking the Gly-Gly-Ala repeats region		Has an enhanced ability to produce recombinant protein compared to HEK293-EBNA	
HEK293FTM	Derived from 293 cells by stable transfection of an FRT-site containing plasmid and of a TetR expression plasmid.		Used for fast and easy generation of a stably transfected cell pool by co-transfecting a Flp-InTM expression vector containing a gene of interest and a Flp recombinase expression vector.	Before 2001
HEK293SG	Ricin toxin-resistant clone derived from HEK293S by ethylmethanesulfonate (EMS).	Yes	Lacks N-acetylglucosaminyltransferase I activity (encoded by the *MGAT1* gene) and accordingly predominantly modifies glycoproteins with the Man5GlcNAc2 N-glycan. HEK293SG is used to produce homogenously N-glycosylated proteins	2001-2002
HEK293SGGD (Glycodelete)	Derives from 293SG through expression of a Golgi targeted form of endoT, an endoglycosidase from the fungus *Trichoderma reesei*		HEK293SGGD is mainly used to produce proteins for glycosylation studies and structural analysis	2010
HEK293A	Subclone of the HEK293 cells with a relatively flat morphology		Facilitates the initial production, amplification and titering of replication-incompetent adenovirus	
HEK293MSR	Genetically engineered from HEK293 to express the human macrophage scavenger receptor		Strongly adheres to standard tissue culture plates for dependable results	

Data adapted from [9,10].

**Table 2:** Genes that have been engineered to modulate proliferation in the HEK293 cell line.

Gene	Function	Modification	Outcome	Recombinant Protein(s)	Reference
Cyclin-dependent kinase like 3 (*CDKL3*)	Promotes cell cycle G1-S transition in mammalian cells	Overexpression	19% improvement in growth rate, 20% improvement in r. protein production, 8% greater maximum viable cell density	Secreted recombinant adipocyte complement-related protein of 30kDa	[56]
Cytochrome c oxidase subunit 15 (*COX15*)	Counteracts apoptosis due to its involvement in the Synthesis of heme		13% improvement in growth rate and 11% improvement in r. protein production		
Eukaryotic initiation factor 3 subunit i (***EIF3I***)	Subunit of the eIF3 targets and initiates translation of a subset of mRNAs involved in cell proliferation, including cell cycling, differentiation, and apoptosis.	Overexpression	Faster growth and increased c-Myc expression.	Renilla and Firefly luciferase	[57]
Cyclin-dependent kinase inhibitor 1B (***CDKN1B***)	Binds to and prevents the activation of cyclin E-CDK2 or cyclin D-CDK4 complexes, and thus controls the cell cycle progression at G1	Inducible over-expression	5.9-fold increase in recombinant protein expression	Recombinant secreted alkaline phosphatase (SEAP)	[59]
Cyclin-dependent kinase inhibitor 1A (***CDKN1A***)	Binds to and inhibits the activity of cyclin-cyclin-dependent kinase2 or cyclin-dependent kinase4 complexes, and thus functions as a regulator of cell cycle progression at G1	Overexpression (Coupled with rational vector design and valporic acid treatment)	Approximately 27-fold increase in recombinant protein production, from 40 mg/L to 1.1 g/L in transient system	IgG	[58]
Cyclin-dependent Kinase inhibitor 2C (***CDKN2C***)	Interacts with CDK4 or CDK6, and prevent the activation of the CDK kinases, thus functioning as a cell growth regulator that controls cell cycle G1 progression				
Acidic fibroblast growth factor (*FGF1*)	Involved in a variety of biological processes, including embryonic development, cell growth, morphogenesis, tissue repair, tumor growth and invasion				

**Table 3:** Genes that have been engineered to delay apoptosis in the HEK293 cell line.

Gene	Function	Modification	Outcome	Recombinant Protein(s)	Reference
X-linked inhibitor of apoptosis (***XIAP***)	Inhibits the apoptosis executioners caspase3, caspase 7, and caspase 9	Overexpression	8-fold higher number of viable cells., extension of stationary phase by 48 h	N/A	[62] [63]
B-cell lymphoma 2 (***BCL2***)	Inhibits the pro-apoptotic proteins Bak and Bax	Overexpression Over-expression	Extension of stationary phase by 48 h 53% increase in volumetric production	N/A Lentiviral vectors	[63] [66]
Caspase 3 (*CASP3*) , Caspase 6 (*CASP6*) , Caspase 7 (*CASP7*) and Allograft inflammatory factor 1 (***AIF1***)	Executioners of apoptosis in the caspase dependent pathway	Quadruple Knockout	Higher expression levels of r. proteins and higher packaging efficiency of recombinant viral particles	Bax, TRAIL, Luciferase Lentivirus	[61]
Bcl-2-associated X protein (***BAX***) and BCL2 Antagonist/Killer 1 (***BAK***)	Activation of caspase proteins	Double knockout	Resistance to apoptosis and sheer stress, 40% improvement in recombinant protein titers	Human IgG1 antibody	[60]
***CrmA*** (viral)	Apoptosis inhibitor	Overexpression	Resistance to numerous apoptotic insults	N/A	[64]
Nuclear factor erythroid 2-related factor 2 (***NRF2***)	Transcription factor that upregulates antioxidant response elements (AREs)-mediated expression of antioxidant enzyme and cytoprotective proteins.	Overexpression	Higher growth rate, more resistant to oxidative stress. 1.7-fold improvement in r. protein expression	Recombinant coagulation factor VII	[65]

**Table 4:** Genes that have been engineered to improve central carbon metabolism in the HEK293 cell line.

Gene	Function	Modification	Outcome	Recombinant Protein(s)	Reference
Pyruvate Carboxylase (*PC*)	Catalyzes the conversion of pyruvate to oxaloacetate	Overexpression	Increased maximal cell density, viability, and glucose utilization.	Adenovirus	[77]
			Decreased lactate and ammonia production.	Recombinant Interferon α2b	[78]
			Improved glucose metabolism.		[79]
			Improved total volumetric recombinant protein production		[80]
Pyruvate dehydrogenase kinase (*PDK*)	Inactivates the pyruvate dehydrogenase enzyme complex, increasing the conversion of pyruvate to lactate in the cytosol.	Knockdown	30-fold increase in specific productivity of infectious viral particles and a 4-fold decrease in lactate production	Retroviral vectors	[81]
Hypoxia inducible factor 1 (*HIF1A*)	Activates an over-expression cascade of glycolytic enzymes and glycolysis-related genes, including pyruvate dehydrogenase kinase (*PDK*) and lactate dehydrogenase (*LDH*)	Knockdown			

**Table 5:** Genes that have been engineered to improve glycan pattern homogeneity and glycosylation efficiency in the HEK293 cell line.

Gene	Function	Modification	Outcome	Recombinant Protein(s)	Reference
*N*-acetylglucosaminyltransferase I (GnTI) protein encoded by the ***MGAT1*** gene	Processing of high-mannose to hybrid and complex N-glycans	Knockout	Inability to synthesize complex glycans. Only the Man5GlcNAc2 N-Glycan present	Rhodopsin	[87]
Endo-β-N-acetylglucosaminidase from *Hypocrea jecorina (endoT^8^)* fused to the Golgi targeting domain of the human β-galactoside-α-2,6-sialyltransferase 1 (in GnTI-/- cells)	Catalyzes the hydrolysis of mannose modifications to produce free oligosaccharides. Catalyzes the transfer of sialic acid from CMP-sialic acid to galactose-containing substrates	Overexpression	Improved homogeneity in glycan expression	Granulocyte macrophage colony-stimulating factor (GM-CSF), monoclonal anti-CD20 antibody GA101	[90]
*Golgi mannosidases* *MAN1A1*	Catalyzes the removal of 3 distinct mannose residues from peptide-bound Man (9)-GlcNAc (2) oligosaccharides.	Triple knockout	Production of simple Man9GlcNAc2 and Man8GlcNAc2 structures of high-Man–type glycans; limited complex-type N-glycans at relatively low abundance	Lysosomal enzymes, α-galactosidase-A (GLA) and lysosomal acid lipase (LIPA)	[89]
*MAN1A2*	Progressively trim alpha-1,2-linked mannose residues from Man(9)GlcNAc(2) to produce Man(5)GlcNAc(2).				
**ER mannosidase** *MAN1B1*	Plays an important role in the disposal of misfolded glycoproteins				
***MAN1C1*** and ***MGAT1*** (quadruple and quintuple knockouts based on the triple knockout by Jin and colleagues [89]	Trim alpha-1,2-linked mannose residues from Man(9)GlcNAc(2) to produce first Man(8)GlcNAc(2) then Man(6)GlcNAc and a small amount of Man(5)GlcNAc. Essential for the conversion of high-mannose to hybrid and complex N-glycans	Quadruple and quintuple knockouts	Elimination of all the hybrid-type and complex-type N-glycans with only the high-mannose-type N-glycans present	Lysosomal acid lipase (LIPA) and immunoglobulin G1 (IgG_1_)	[86]
Sialyl transferases, *ST6GAL1*, *ST3GAL3* and *ST3GAL4* *ST6GAL1*	Add sialic acid to the terminal portions of the N- or O-linked sugar chains of glycoproteins.	Overexpression Overexpression	Enhanced sialylation Increased α2,6 sialylation	EPO Trastuzumab F243A mutant antibody	[91] [92]
Golgi mannosidases ***MAN2A1*** and ***MAN2A2***	Control conversion of high mannose to complex N-glycans	Double Knockout	Hybrid-type N-glycans only	Lysosomal acid lipase (LIPA) and Fc of immunoglobulin G1 (IgG_1_)	[88]
Fucosyltransferase 8 (***FUT8***)	Catalyzes the addition of fucose in alpha 1-6 linkage to the first GlcNAc residue, next to the peptide chains in N-glycans.	Triple knockout with MAN2A1 and MAN2A2	Hybrid-type N-glycans only without core fucosylation	Lysosomal acid lipase (LIPA) and Fc of immunoglobulin G1 (IgG_1_)	[88]

**Table 6:** Genes that have been engineered to improve recombinant protein folding in the HEK293 cell line.

Gene	Function	Modification	Outcome	Recombinant Protein	Reference
Human resistance to inhibitors of cholinesterase 3 (***RIC3***)	Molecular chaperone of nicotinic acetylcholine receptors	Overexpression	Expression of high levels of functional receptor protein	α7 nicotinic acetyl cholinesterase receptor	[95]
X-box binding protein (***XBP1***)	Induces the unfolded protein response by upregulating target genes encoding ER chaperones and ER-associated degradation (ERAD) components to enhance the capacity of productive folding and degradation mechanism, respectively.	Overexpression	Improvement in volumetric and cell specific gamma retroviral productivity	Gamma retroviral vectors	[66]
Protein Disulfide Isomerase Family A Member 2 (***PDIA2***)	Folding of nascent proteins in the endoplasmic reticulum by forming disulfide bonds through its thiol isomerase, oxidase and reductase activity				
Heat shock protein family A (Hsp70) member 5 (***HSPA5***)	Plays a key role in protein folding and quality control in the endoplasmic reticulum lumen. Involved in the correct folding of proteins and degradation of misfolded proteins.				
Vitamin K Epoxide Reductase Complex Subunit 1 **(*VKORC1***)	Carboxylation of glutamic acid residues in some blood-clotting proteins, including factor VII, factor IX, and factor X	Overexpression	Improved γ-carboxylation	rhFVII	[97]
Calumenin (*CALU*)	Inhibitor of γ-carboxylase	shRNA inhibition	Up to 7.5-fold increase in production yield of active enzyme	rhFX	[96]

**Table 7:** Genes that have been engineered to improve the secretory machinery of the HEK293 cell line.

Gene	Function	Modification	Outcome	Recombinant Protein	Reference
SNARE receptor coding genes, ***SNAP-23*** and ***VAMP8***	Mediate/catalyze the fusion of vesicles to target membranes during exocytosis	Overexpression	Improved recombinant protein expression	SEAP, SAMY	[110]
SM protein coding genes *MUNC18* and *s**ly1***	Regulate SNARE complex assembly and then cooperate with SNAREs to stimulate membrane fusion.				
Tetraspanin ***CD9***	Play a role in many cellular processes including exosome biogenesis.	Overexpression	Up to 26% improvement in virus production	Exosome-Associated AAV	[109]

**Table 8:** Whole genome analyses and engineering strategies for the identification of engineering gene targets for improved recombinant protein expression in the HEK293 cell line.

Type of Screen/Analysis	Genes/Genomic Elements/ Pathways Identified	Recombinant Protein(s)	Ref.	Genes/Genomic Elements Identified or Confirmed in Follow-up Study(ies)	Modification	Outcome	Ref.
miRNA	hsa-miR-22-5p, hsa-miR-18a-5p, hsa-miR-22-3p, hsa-miR-429 and hsa miR-2110	Neurotensin receptor, cytoplasmic luciferase and secreted glypican −3 hFc-fusion protein (GPC3)	[111]	*HIPK1*	Knockdown	3.2-fold higher Luciferase and 2.3-fold higher GPC3 expression	[112]
				miRNA-22-3b	Stable overexpression	2.4-fold higher luciferase activity	[113]
				*HIPK1*	Knockout	4.7-fold higher luciferase expression	
siRNA	CCAAT/enhancer binding protein gamma (*CEBPG*)	Recombinant EPO, interferon γ and monoclonal antibody	[114]				
siRNA	*INTS1, HNRHPC, CASP8AP2, OAZ1* and *PPP2R1A*	Luciferase, GPC3, neurotensin receptor type I and serotonin transporter	[115]	*OAZ1*	Knockout	5-fold higher specific luciferase and 2.5-fold higher specific SEAP production	[116]
				*CASP8AP2*	Knockout	7-fold increase in specific luciferase and 2.5-fold increase in specific SEAP expression	[117]
Multi-omics analysis (transcriptomic, fluxomic, metabolomic)	*FABP5, RETNLB,* pyruvate carboxylase (PC) X-box binding protein (*XBP1*), *HSPA5* and *HERPUD1*	A heavy chain variable region fused to the Fc region of a human IgG (dAb-Fc)	[118]				
Transcriptomic analysis	Amino acid metabolism, carbohydrate metabolism, polyamine metabolism, glutathione metabolism, nucleotide metabolism, the pentose phosphate pathway and lipid metabolism	A murine leukemia virus-based vector.	[119]				
Proteomic analysis	*NEDD8, NEDD4L, ESCRT1*, lipid biosynthesis, glycosphingolipids metabolism, calcium regulation, oxidant detoxification, xenobiotic metabolism, peptidase activity, DNA detoxification, endocytosis and late endosomal-related pathways	HIV-gag VLP	[120]	*NEDD8*	Overexpression	1.5-fold increase in VLP production.	[121]
				*NEDD4L*	Overexpression	3.3- fold increase in VLP production.	
				*UGCG*	Overexpression	2.9- fold increase in VLP production.	
				*CIT*	Overexpression	2.4-fold increase in VLP production.	
				*CNP*	ShRNA knockdown	2.7-fold increase in VLP production	
